# Efficient Detection of Knee Anterior Cruciate Ligament from Magnetic Resonance Imaging Using Deep Learning Approach

**DOI:** 10.3390/diagnostics11010105

**Published:** 2021-01-11

**Authors:** Mazhar Javed Awan, Mohd Shafry Mohd Rahim, Naomie Salim, Mazin Abed Mohammed, Begonya Garcia-Zapirain, Karrar Hameed Abdulkareem

**Affiliations:** 1 School of Computing, Faculty of Engineering, Universiti Teknologi Malaysia (UTM), Johor 81310, Malaysia; shafry@utm.my (M.S.M.R.); naomie@utm.my (N.S.); 2 Department of Software Engineering, University of Management and Technology, Lahore 54770, Pakistan; 3 College of Computer Science and Information Technology, University of Anbar, 11, Ramadi, Anbar 31001, Iraq; mazinalshujeary@uoanbar.edu.iq; 4 eVIDA Lab, University of Deusto, 48007 Bilbao, Spain; 5 College of Agriculture, Al-Muthanna University, Samawah 66001, Iraq; khak9784@mu.edu.iq

**Keywords:** anterior cruciate ligament, healthcare, knee injury, artificial intelligence, convolutional neural network, MRI, detection, classification, residual network, augmentation

## Abstract

The most commonly injured ligament in the human body is an anterior cruciate ligament (ACL). ACL injury is standard among the football, basketball and soccer players. The study aims to detect anterior cruciate ligament injury in an early stage via efficient and thorough automatic magnetic resonance imaging without involving radiologists, through a deep learning method. The proposed approach in this paper used a customized 14 layers ResNet-14 architecture of convolutional neural network (CNN) with six different directions by using class balancing and data augmentation. The performance was evaluated using accuracy, sensitivity, specificity, precision and F1 score of our customized ResNet-14 deep learning architecture with hybrid class balancing and real-time data augmentation after 5-fold cross-validation, with results of 0.920%, 0.916%, 0.946%, 0.916% and 0.923%, respectively. For our proposed ResNet-14 CNN the average area under curves (AUCs) for healthy tear, partial tear and fully ruptured tear had results of 0.980%, 0.970%, and 0.999%, respectively. The proposing diagnostic results indicated that our model could be used to detect automatically and evaluate ACL injuries in athletes using the proposed deep-learning approach.

## 1. Introduction

**Figure 1:**
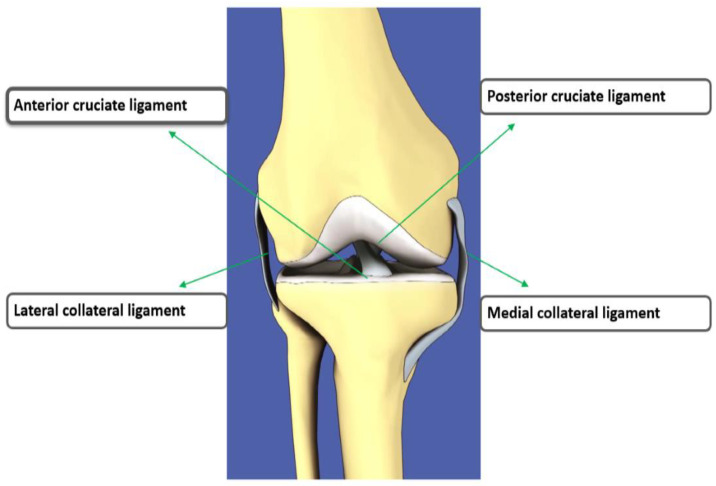
The front view of the 4 major ligaments of knee anatomy, where the anterior cruciate ligament (ACL) is located at the center of the knee, the posterior cruciate ligament at back of the knee, the medial collateral ligament at the inner knee and the lateral collateral ligament at the outer knee [2].

The anterior cruciate ligament (ACL) is an important stabilizing ligament of the knee that connects the femur to the tibia [1]. In the knee, there are four primary ligaments: two ligaments inside the knee are anterior cruciate ligament, posterior cruciate ligament while two outside ligaments are lateral collateral ligament, medial collateral ligament. Figure 1 shows the anatomy of knee ligament tears [2]. The ACL is the most common injured knee ligament in athletes. It provides the stability as the knee moves. This movement can produce increased friction on the meniscus and cartilage in the joint. The symptoms of ACL include pain, swelling and deformation of the knee, making walking difficult [3,4]. A radiologist’s work is to detect various injuries, such as torn ACLs from radiological scans. It is a time-consuming process to interpret knee ACL injuries, tears in meniscus, knee cartilages abnormalities which causes knee osteoarthritis, osteoporosis and knee joint replacement from radiology images manually [5]. There are many methods to diagnose an ACL tear in the knee: physical tests, and biomarkers [6], X-ray, computed tomography (CT), mammography, ultrasound imaging and magnetic resonance imaging (MRI) [7]. MRI is the best choice for diagnosing ACL tears as ACL is not visible as a plain file X-ray [8,9,10].

MRI can distinguish sprains and partial tears of the ACL from complete as well as meniscus tears [11]. Typically, an ACL is a low band of signal intensity traversing from the femoral end to the turbulent either seen totally in one single slice or multiple slices depending on the obliquity of the scanning done. The ACL tear has to be read in sequence of coronal, sagittal and axial planes to give the whole idea about ACL tear [12]. The three grades areas shown in Table 1.

In recent years, the machine learning and deep learning methods for image analytics have been extensity used in the medical imagining domain to solve the problems of classification, detection, segmentation, diagnosis without involvement of radiologist [13,14,15,16]. Nowadays, researchers are using deep learning with a model of CNN and its architectures in several applications. The CNNs architectures have an input layer and an output layer, and there are also many convolutional layers, pooling layers, rectified linear unit layers, dense layers and dropout layers [17,18]. The CNN shows huge success in the analysis of radiography X-rays in the knee osteoarthritis automatically, as there is no need of image pre-processing [19,20]. However, X-rays have not been able to improve upon three classes of knee ACL detection, as compared to MR images.

This study aims to further enhance the automatic performance, without involving a radiologist, by using a deep learning model to detect the anterior cruciate ligament by an inspecting MRI. The customized residual network (ResNet-14) architecture of CNN is proposed in the study, and it has significantly improved the detection of healthy, partially and completely ruptured ACL tears. Here, we train our modified model on 6 different approaches which have achieved promising results on the KneeMRI data set. The two strategies: hybrid class balancing and real time data augmentation were taken to address the KneeMRI scarcity and class imbalance issues in this study.

Our study has the following contributions that is summarized as below:

- To the best of our knowledge, this study is the first that propose a balancing methodology for three classes healthy, partial, and ruptured tears based on hybrid class balancing and real-time data augmentation.
- This study propose a customized ResNet-14 CNN model without transfer learning to detect three classes of ACL.
- We perform an extensive experimental validation of the proposed approaches in term of sensitivity, specificity, precision, recall, F1- measure, receiver operating curve (ROC), area under curve (AUC).

The remainder of the paper is arranged as follows: Section 2 discusses related work. Section 3 explains the details of the data set and proposed methodology of the model and architecture. The results of our experimental evaluation is presented in Section 4. Section 5 related to discussion of our work compared with state of art work. Finally, Section 6 related to conclusion.

## 2. Related Work

There is a growing body of literature in the knee bone MRI detection. Numerous researchers are working at their best using machine learning and deep learning techniques to identify the disease through MR images in better and novel ways. The study [21] has shown good results, after using support vector machines on 300 MR images of healthy, partial and fully ruptured ACL tears. The study was classified the human articular cartilage OARSI-scored with machine learning pattern recognition and multivariable regression techniques. The regression model was achieved 86% accuracy of normal and osteoarthritic [22]. The first real attempt was related to our dataset of the KneeMRI [23] through techniques of feature extraction, histogram-oriented gradient (HOG) descriptor and gist descriptor manually. The performance of ACL tear was measured by the AUC for the injury-detection 0.894 problem and for full rupture case 0.943 after being coupled with both features and machine learning support vector machines (SVM) and random forest (RF). There are various surveys, meta-analyses and reviews [24,25] related to anterior cruciate ligament knee injury detection through various machine learning models. It has been shown that the accuracy remained good in the case of a smaller dataset, but in the case of more radiology images, the machine learning models have not been a solution. The machine learning cannot be a very useful solution for diagnosis and detection, particular in the case of knee injury.

The authors (Manna, Bhattacharya et al. 2020 [26]) proposed a self-supervised approach with pretext and downstream tasks using class balancing through oversampling showed accuracy of 90.6% to detect ACL tear from knee MRI.

The state-of-the-art-work [27] related to deep learning was presented as AlexNet [28] architecture of convolutional neural network (CNN) to extract features of knee MRNet with transfer learning ImageNet [29]. The performance of these dataset found AUC 0.937, 0.965 and 0.847 of abnormalities, ACL tears and meniscus tears respectively, whereas in the case of external validation KneeMRI dataset the AUC was 0.911. The results were better as compared to the semi-automated earlier work of KneeMRI [23] for ACL tear detection in the case of machine learning. The study proposed multiple CNN architectures using U-Net [30] and Res-Net [31] to detect complete anterior cruciate ligament tear from dataset FastMRI [32]. The accuracy of cropped images found 0.720, cropped with dynamically 0.765 and for uncropped images that were found 0.680 only [33].

In a previous study, Liu et al. [34] proposed hybrid architectures of CNN to detect ACL tears. Firstly, the authors used architecture LeNet-5 [35] to detect slice detection of ACL; secondly, they extracted an intercondylar notch in the ACL part using you only look once (YOLO) [36] and lastly, they adopted the densely connected convolutional network DenseNet [37] to classify the presence or absence of an ACL tear with an AUC 0.98. The classification is also determined through (VGG16) [38] and AlexNet with AUC 0.95 and 0.90, respectively. However, the burden of training the all three architectures, in a cascaded fashion, is computationally expensive and time consuming. In the study, Namiri et al. [39] used 3D CNN classify hierarchical severity stages in ACL automatically, that had an accuracy 3% more than 2D CNN. The study of [40] related arthroscopy findings of MRI dataset and used DenseNet architecture upon 489 MRI samples only, in which 163 were from an ACL tear and 245 were from an intact ACL. The comparison study related to musculoskeletal Irmakci et al. [41] performed three CNN architectures AlexNet, ResNet and GoogleNet, that achieved AUC 0.938, 0.956 and 0.890, respectively, detecting ACL tears on MRNet dataset. The ResNet-18 model was found better in the case of an ACL tear, but in the case of abnormalities, the ResNet result was not good. The challenging task was a meniscus tear with low accuracy and in terms of sensitivity as well. The recent state-of-art work [42] used the lightweight model efficiently-layered network ELNet [43] which was evaluated on MRNet with an AUC of 0.960 achieved detecting an ACL tear, and on the KneeMR dataset as well. It evaluated a 5-fold cross-validation to detect injury with AUC of 0.913.

In all the above studies, the authors mostly used knee MRI datasets related to MRNet and KneeMRI. However, in these datasets the classes are not balanced, which causes bias in training data. After using the deep learning architecture, comprehensive training is required in the data. The literature suggests that performances of the area under the curve of ELNet and ResNet were performed with excellent results, as compared to other architectures. Moreover, there are some challenges of detecting the anterior cruciate ligament (ACL) injury currently and efficiently through automated ways without involving radiologist.

## 3. Materials and Methods

This section presents the methods and material used in this study. Section 3.1 details the datasets of MRI images and their features and classes. Next, we will precede to the data pre-processing and class balancing in Section 3.2. Finally, the proposed customized method ResNet is presented and explained using real-time data augmentation in Section 3.3.

### 3.1. Dataset

The total of 917 knees sagittal plane DICOM MRI were obtained from the clinical hospital center of Rijeka [23] archiving and communicating system. Images were 12-bit greyscale color along with assigned ACL diagnosis. An Avanto 1.5T MRI Siemens scanner which manufactured by Muenchen, Germany was used to record all volumes from 2007 to 2010, and for the collection of this data, proton density-weighted fat suppression. The authors have provided the metadata CSV for further understanding in the Table 2. Moreover samples of ACL diagnosis three classes are healthy (0 labels), partial (1 label) and fully ruptured (2 labels) in the Table 2. The total samples are 917 pickle images, out of this 690 are healthy, 172 partials and 55 complete ruptured.

**Figure 2:**
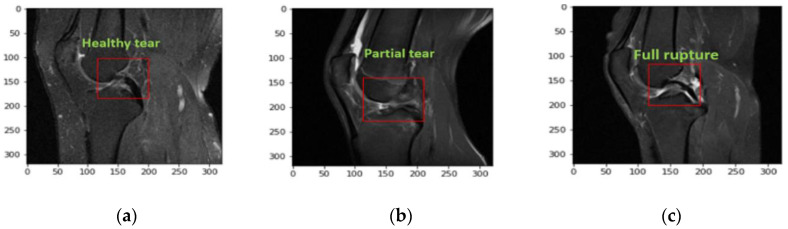
Samples of a healthy tear: (**a**) no changes in the length of an ACL tear, (**b**) sample of an ACL partial tear, (**c**) full rupture in an ACL tear.

The red square in the Figure 2a–c shows the three different severity of ACL tears. These are pickle MRI images of healthy, partial and fully ruptured tears respectively.

### 3.2. Data Pre-Processing

**Figure 3:**
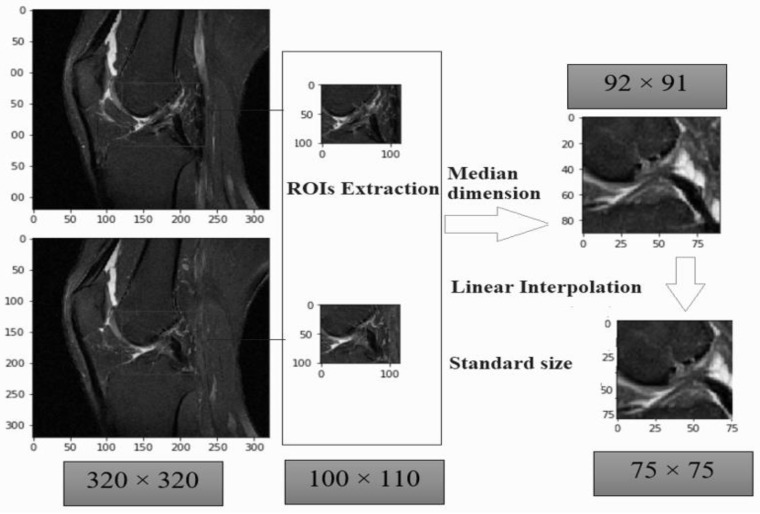
Region of interest-based pre-processing extraction with one standard size.

We performed three steps of data pre-processing on the metadata file and image. As such, we first applied normal approach [44,45] to localize based upon region of interest (ROI). As sample MR images were not of the same widths and heights. The input images were wider ACL area of 290 × 300 × 21 to 320 × 320 × 60 with midmost measurements 320 × 320 × 32. The values were representing slice width, slice height and number of slices respectively in a single volume file. The ROIs focused on a region or subset of tissues in the MRI slices and get rid of unnecessary details from the inspected images. The ROIs boundary were calculated manually sum of ROIY axis with ROI height value and sum of ROIX axis with ROI width columns present in our metadata file or in Table 2. For this way the ROIs obtained various dimensions from 54 × 46 × 2 to 124 × 136 × 6, having average dimensions 92 × 91 × 3. All the ROIs were varied in size which can affect our training as well. We rescaled all the ROIs slices using linear interpolation to fix one standard size of 75 × 75. This rescaling can enhance our model performance in Google Colab but there was also problem of lossless of visual features exists in some slices. The Figure 3, illustrates where the sample input image with dimensions of 320 × 320 × 60. The median dimension of an extracted ROI is 92 × 91. The standard size of all ROI was fit into the dimension of 75 × 75.

Secondly, before feeding our dataset into our model, we need to map our extracted ROI with the corresponding labels that we have extracted from the structured data file.

Lastly we handled the problem of class balance through a hybrid approach with over-sampling and under-sampling. Thus, there are total 3081 pickle MRI images initially, which consisted of: healthy tears (2315 images), partial tears (580 images) and fully ruptured tears (186 images). There is problem of class imbalance in terms of distribution among three classes. The under-sampling technique is reduced the number of samples from the majority class to match up the total length with minority class samples. This technique is not generalized on unseen data, so there is a chance of information loss, biased sample and not given the accurate representation of the whole sample. For this we excluded random under-sampling in the label 0 majority class and added randomly more observations by replication in our minority classes of label 1 and label 2.The under-sampling is only preferred when the minority class sample is high. On the other hand, the over-sampling technique is increased the number of samples in the minority class to match up the number of samples in the majority class but it caused of over-fitting [46,47,48].

**Figure 4:**
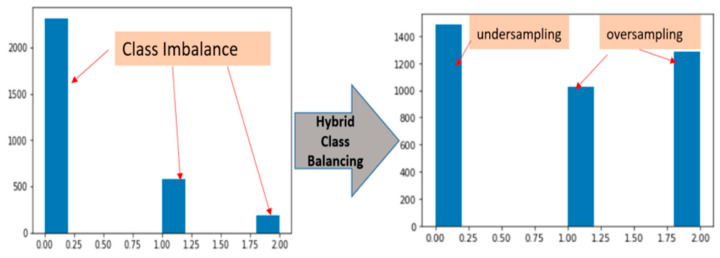
Hybrid class balancing with under-sampling and over-sampling.

Figure 4 shows the hybrid class balancing, the bars of each class becoming almost equally distributed. After the hybrid class balancing the sample size of three classes are raised. The new values are now 1487, 1027 and 1283 of healthy, partial and full ruptured tears respectively.

### 3.3. Our Proposed Custom ResNet-14 Architecture

**Figure 5:**
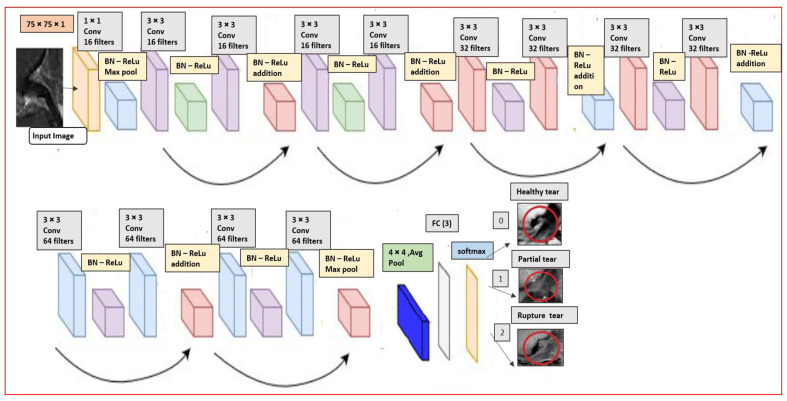
Our customized ResNet-14 architecture.

In this section we will briefly explain the proposed CNN custom Residual ResNet architecture. After all the pre-processing steps above the authors have built an end-to-end model by modifying the original version-I residual ResNet-18 [31], into proposed ResNet-14 network structure as it illustrated in Figure 5. The MR image with dimension 75 × 75 × 1 is provided as input layer in the structure. We added batch normalization (BN) [49] in the model before the activation function rectified linear unit (Relu) and right after convolutional layers (Conv) with 3 × 3, which acts like a regularization. The vanishing gradient problem is reduced significantly through this operation. In addition to this, a sequence of 3 inner ResNet stacks of convolutional with stride 2 of max pooling 3 × 3 with *n* = 2 parameters instead of 3 to avoid the overfitting. There are totally 6*n* + 2 stacked weighted layers.

Further, we are used to controlling the learning process with fine-tuned hyper-parameters by manually having a great impact on the performance of the model. In the complied stage on the proposed architecture, we have chosen the Adam [50] optimizer, which can keep tracks of an exponentially decay average. The learning rate was configured to be set dynamically on the basic of the number of epochs, batch size to 32 and the learning rate is 0.001 as in our case we used with 120 epochs. At the ends, 3 fully connected layers (FC) with average pooling (Avg pool) and softmax activation function have been added to detect the healthy, partial and rupture tears in the MRI. The details of the convolutional layers and their order in the custom ResNet-14 model in the Table 3. The total number of parameters are 179,075.

Finally we involved the real-time data augmentation in our model, which generated different images after running each epoch. It randomly augmented the image at runtime and applied transformation in mini-batches [51]. So, it is more efficient than offline augmentation because it does not require extensive training. The technique of offline data augmentation significantly increased the diversity of their available data without actually collecting new data by cropping, padding, flipping, rotating and combining in the case of Alzheimer’s stage detection, brain tumor and others in the MRI [52,53,54].

The real-time data augmentation performed good accuracy with the CNN inception v3 model for breast cancer [55]. We used real time data augmentation with a class Image_Data_generator which generated batches of tensor image data [56,57,58] from the keras library. The following Table 4, describes about augmentation parameters which we used in the real time augmentation.

**Figure 6:**
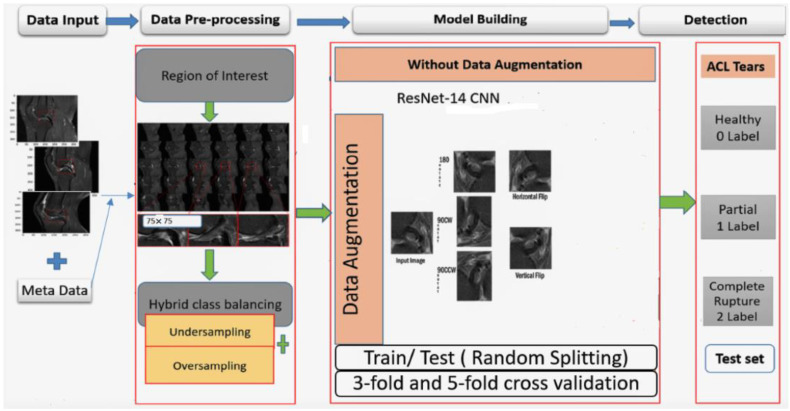
A block diagram of the proposed methodology.

Furthermore, the block diagram of the proposed work’s whole process is illustrated in Figure 6, with four main stages. Firstly, the data input stage, where the image dimension is combined with metadata to generate images through the pickle library. In the second stage, the images are resized through the region of interest and then applied with hybrid-class balancing. The model building stage is done through our custom ResNet-14 with and without online data augmentation. In the last stage, the performance is measured and compared through random train/test split and K-fold cross-validation to detect anterior cruciate ligament tear.

## 4. Experimental Results

In this section we will present the experimental setup, to analyze our model and to evaluate the results.

### 4.1. Experimental Setup

The experiments were carried out on Google Colab with Python 3.6. The paper [59] in which the CNN model was implemented on knee cancellous bones achieved 99% accuracy, with better acceleration. So we selected Google Colab, providing free GPU, with the specifications of the Tesla K80 processor having 2496 CUDA cores and 12GB ram. The ResNet Model is coded by using Keas (version 1.0) backend Tensor Flow. The model has been validated with train and test split and cross-validation techniques.

### 4.2. Train/Test Split

**Figure 7:**
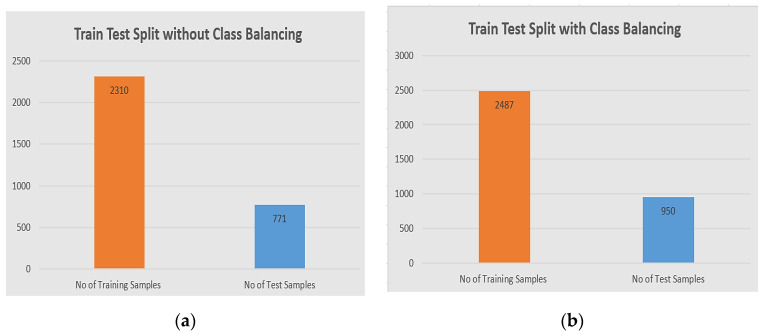
The division of train/test validation: (**a**) the distribution of training samples are 2310 in the training set, and 771 in the test set original dataset before class balancing; (**b**) the distribution of training samples is 2387, and the test is 950 after class balancing.

The model has been validated through the train and test split, for each approach with and without class balancing, and at the same time we have to split our full dataset into X train and Y test after image normalization. We used 75% of the total data for training purposes and 25% for testing purposes. We have used two samples before class balancing and after class balancing. The detail of the train test split division is shown in the Figure 7.

### 4.3. K- Fold Cross-Validation

The model has been validated in K-fold cross-validation, the data is randomly divided up into K groups known as folds. One of those folds is kept as the validation set, and the remaining data is used for the training. The mean loss from all the folds is the overall K fold loss. Same as loss, the average of accuracy from all the folds is the overall accuracy. We used techniques for this is train/test split cross-validation with K = 3 and K = 5. The k- fold cross-validation has been reduced the bias, and the variance is reduced after each k folds.

In order to evaluate performance of our model, we measured through the confusion matrix where the measurement criteria were precision, sensitivity, F1-score, specificity and weighted average. We considered the receiver operating characteristic (ROC) curve and area under curve (AUC).

### 4.4. Prediction Performance of Proposed ResNet

We complied to set the prediction of our model with the parameters cross-entropy loss function, Adam optimizer with a learning rate of 0.001, the number of batch sizes are 32 and the number of epochs for training the model used here was 120. Table 5 shows the test loss and test accuracy after fitting the model of 120 epochs. Moreover, we evaluated and tested our model of ResNet CNN with six different approaches, as mentioned in Table 5.

The minimum loss value of 0.466 is the best approach for our model, which is after class balancing, augmentation with 5-fold cross-validation. The accuracy is computed by dividing the number of correct predictions by the total number of predictions made and then multiplying by a hundred to get the percentage. We also tested result with accuracy of all six approaches whereas the model ResNet-14 with class balancing data augmentation achieved 92% through 5-fold fold cross validation. The detail of the performance of each approach is shown in the Table 6.

## 5. Discussion

In this study, we demonstrate in detail a fully automated ACL detection with the related work. We study the problem of efficient detection of ACL and the accurate selection of the ROI boundaries using the deep learning-based custom Residual Network of 14 layers CNN. We compare the performance of a ResNet-14 with and without class balancing and data augmentation as explained in Table 6. When we applied the model without class balancing the overall accuracy remained under 80.5% for detecting healthy, partial and ruptured tears. There was no significant difference in the accuracy in the case of hybrid class balance data augmentation with random splitting and k-fold cross validation. However, the highest accuracy is observed with hybrid class balancing using data augmentation of ResNet-14 CNN model of 92%.

**Figure 8:**
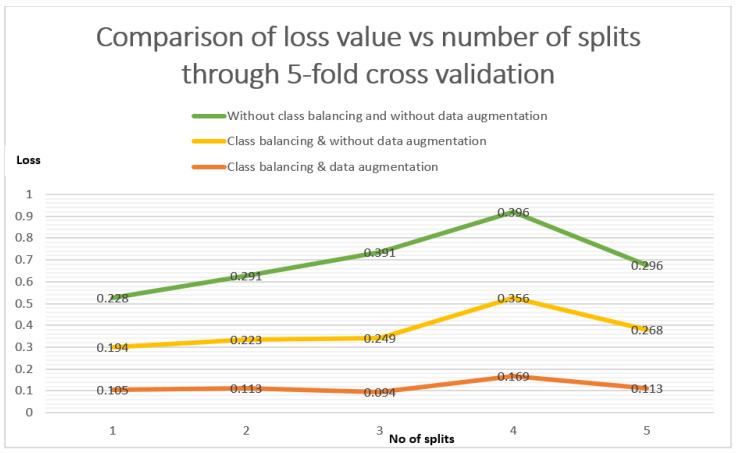
Loss comparison of class balancing and augmentation through 5-fold cross validation.

The three approaches are, (1) without class balancing and data augmentation, (2) class balancing without data augmentation, and (3) class balancing and data augmentation. There are the comparison of three approaches in between loss values vs. each split. The orange line is related to our standard approach of class balancing and with data augmentation in Figure 8. It is illustrated that the error loss value in the case of 1-split is 01.05, and that remained less than the other two approaches even after the 5-split is 0.113.

**Figure 9:**
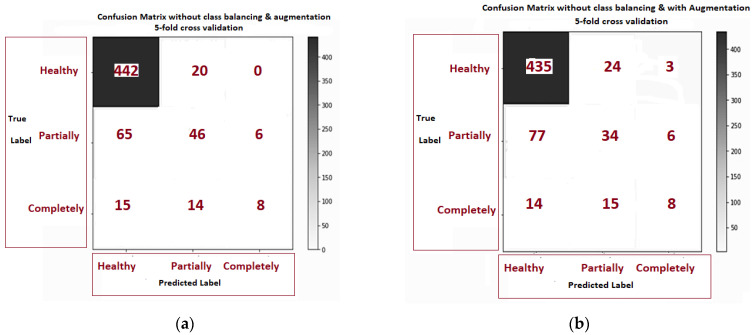

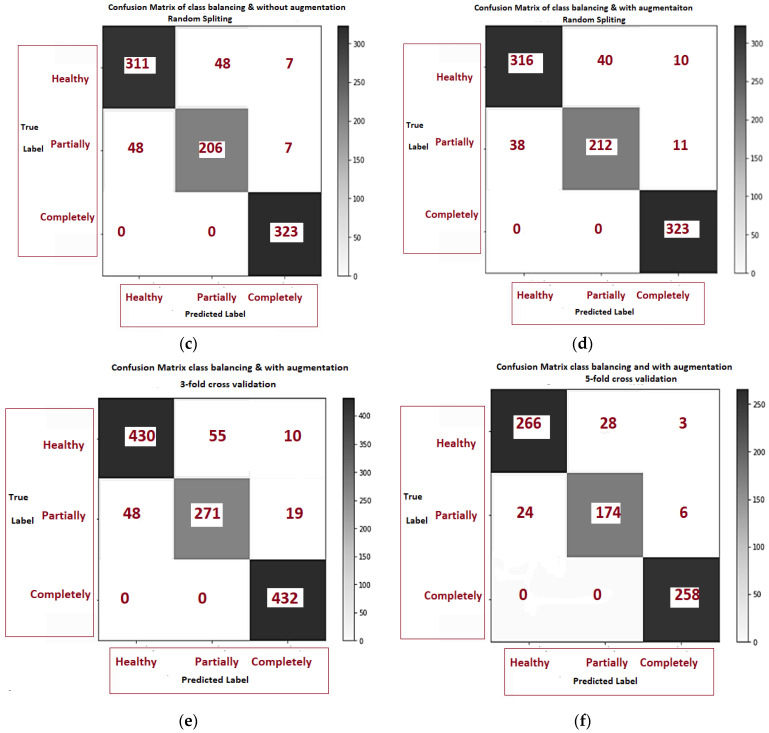
The confusion matrix comparison of six approaches class balancing and data augmentation: (**a**) without class balance and augmentation 5-fold cross validation; (**b**) without class balance but augmentation 5- fold cross validation; (**c**) class balance without augmentation; (**d**) class balance but augmentation; (**e**) class balance and augmentation 3-fold cross validation (**f**) class balance and augmentation 5-fold cross validation.

**Figure 10:**
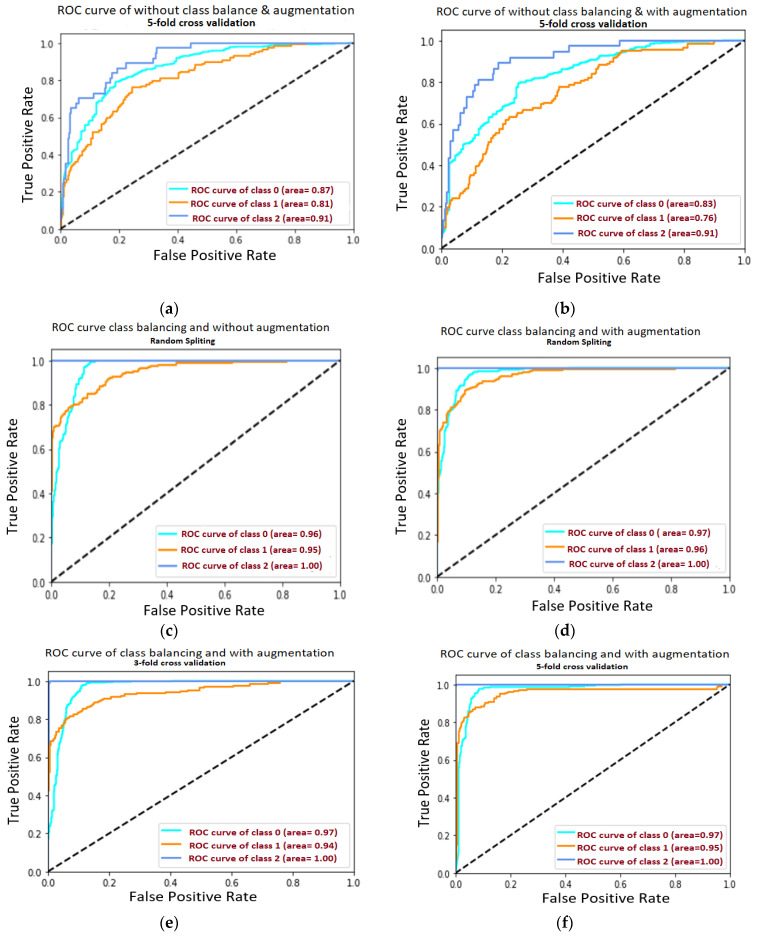
The area under curves of the ResNet CNN Model. (**a**) The area under curve of each class after 5-fold cross-validation in the case without class balancing and without data augmentation where the average AUC is 0.863. (**b**) The area under curve of each class after 5-fold cross-validation in the case without class balancing but with data augmentation of AUC 0.833. (**c**) The area under curve of each class after random train/test split in the case of hybrid class balancing but without data augmentation, the AUC is 0.966. (**d**) The area under curve after random train/test split in the case of hybrid class balancing and also apply data augmentation of AUC 0.973 (**e**) The area under curve 3-fold cross-validation in the case of hybrid class balancing and with data augmentation of AUC 0.966. (**f**) The area under curve after plot 5-fold cross-validation in the case of hybrid class balancing and with data augmentation and AUC is highest 0.98 from all approaches.

Figure 9a–f is related to the confusion matrix of all six approaches with true positive, true negative, false positive and false negative of three classes of healthy, partially and completely ruptured tears. Next, the ROC curves were plotted by computing the true positive ratio (TPR) and false positive ratio (FPR) for six approaches accuracy thresholds as shown in Figure 10 The area under curves of the ResNet CNN Model. Figure 10a–f. From this, the proposed ResNet-14 with hybrid class balancing and data augmentation managed to achieve an area under curve of the ROC curve (AUC) average of 98%.

Eight groups have previously used deep learning methodology to detect ACL tears of various pathology. Table 7 provides a comparison of the performance, datasets and models with our work. The dataset of our work, collected at the Clinical Hospital by Stajduhar et al. [23], related to KneeMRI which showed AUC 0.894 in the case of non-injured cases. These were not recognized well in the case of partial tears. The original MRNet by Bien et al. [27] had no significant change in accuracy in the case of detecting abnormalities and was unable to distinguish in abnormalities because it has taken a tiny portion in 3D imaging. The ACLs full torn sensitivity is 76%, and the AUC was determined as 0.965. For the external data set KneeMRI, it enhanced the AUC 0.911. The ground truth values were not measured correctly by the surgeon. Chang et al. [33] applied the dynamic patch-based residual network to 260 subjects to detect the ACL with accuracy 0.967. However, it had low prevalence in the complete ACL and biased towards high sensitivity due to unbalanced samples. Liu et al. [34] was only considering three CNN models in a cascaded way not a single pipeline which leads the burden of training, no verification of bias, the dataset for training was significantly less. Moreover, it evaluated only on full thickness of ACL tears, not on other classes.

The 3D CNN models were not performed well as compared to 2D CNN due to the small dataset in the work of Namiri et al. [39]. The model was found over-fitting in the case of partial tears, however obtained better results with 3D CNN than with 2D.The sample of patients were not balanced among all grading and dataset split based upon the patients, which caused correlations among multiple images. Lastly, data augmentation techniques were also not applied to enhance the images. The specificity in the case of ACL intact is 88%. Zhang et al. [40] were a long time in the training of each patient, retrospective study inherent biases, the dataset used in this was small, and patient’s category was imbalanced. Moreover, the study did not classify the complete, partial tears of ACL. The study Irmakci et al. [41] was where the average AUC 0.878, 0.857 and 0.859 of models of three classes for AlexNet, ResNet-18 and GoogleNet 0.859 respectively. The one of the state work Tsai et al., 2020 [42] was used EfficientNet which is optimized and in the case of MRNet the AUC was 0.960, but on the knee, MRI AUC was 0.913 due to imbalanced classes.

Zhang et al. [40] took a long time in the training of each patient, with retrospective study inherent biases; the dataset used in this was small and the patient’s category was imbalanced. Moreover, the study did not classify the complete, partial tears of ACL. The study of Irmakci et al. [41] was where the average AUC was 0.878, 0.857 and 0.859 for the models of three classes for AlexNet, ResNet-18 and GoogleNet, respectively. The work of Tsai et al., 2020 [42] used EfficientNet which is optimized and in the case of MRNet the AUC was 0.960, but on the knee, MRI AUC was 0.913 due to imbalanced classes.

## 6. Limitations

Our study had several limitations. First, our ResNet-14 model for ACL tear detection performed individually on all six approaches, which may increase the training burden overall. Secondly, the technique was used for hybrid class balancing, which randomly enhanced the records in the partial tear and fully ruptured tear. The down-sampling in the class label of healthy ACLs in the metadata file was not an appropriate technique, which may have a biased result in the case of the fully ruptured class. The use of class weighting in future studies may further improve the detection performance of the ACL tear detection system. Furthermore, the results were not evaluated on more than 5-fold cross-validation in the case without class balancing.

## 7. Conclusions

This paper has presented an automated system to efficiently detect the presence of anterior cruciate ligament (ACL) injury from MR images in a human knee. The proposed method implements a customized ResNet of 14 layers CNN architecture and has been tested using random splitting, 3-fold cross-validation and 5-fold cross-validation. Using the approach of CNN-ResNet-14, the classes of imbalance distribution was enhanced by hybrid class balancing and the diversity of images was increased without effecting extensive training by applying the real-time data augmentation method. The novel integration of hybrid class balancing and real-time data augmentation operations allow the custom Res-Net model to remain efficient, accurately detect the ACL tears and to avoid the overfitting problem on the KneeMRI dataset. The performance of the CNN customized ResNet-14 with 5-fold cross-validation presents an average accuracy, sensitivity and precision of 92%, 91% and 91% respectively. However, the model achieved a better performance and in the case of the average specificity and AUC for the three classes was 95% and 98%, respectively. In addition, the model has been tested and compared with 3-fold cross-validation and random splitting as well. To the best of the authors’ knowledge, there is no such study that proposes an automated method to detect the anterior cruciate ligament of all three classes of healthy, partial and full ruptured tears through hybrid class balancing of the ResNet-14 model with AUC 98%.

## Figures and Tables

**Table 1:** Three grade stages of the anterior cruciate ligament.

Grade Stages	Injuries/Symptoms
Grade-I	Intra-ligament injury No changes in the ligaments length
Grade-II	Intra-Ligament injury Change in ligament length Partial tears
Grade-III	Complete ligament disruption

**Table 2:** The samples of metadata of 9 features and 1 class label of ACL diagnosis.

Series No	Knee LR	ROIX	ROIY	ROIZ	ROI Height	ROI Width	ROI Depth	Volume Filename	ACL Diagnosis
5	0	126	96	14	78	79	4	502889-5.pck	0
5	0	116	177	13	83	79	4	507277-5.pck	1
5	1	113	140	9	89	96	4	496580-5.pck	2

**Table 3:** The configuration detail of customized ResNet model-14 with their output size.

Layer Name	Output Size	Layer Information
Input layer	75 × 75 × 1	
conv1	75 × 75	1 × 1, strides 2, 16
conv2_d (1 block)	75 × 75	3 × 3, maxpool stride 2 3 × 3, 16
conv2_d (2 block)	75 × 75	3 × 3, 16
conv3_d (1 block)	38 × 38	3 × 3, 32
conv3_d (2 block)	38 × 38	3 × 3, 32
conv4_d (1 block)	19 × 19	3 × 3, 64
conv4_d (2 block)	19 × 19	maxpool 3 × 3, 64
Average Pool	4 × 4	4 × 4 average pool
Fully Connected Layer	Three classes	64 × 3 fully connections
Softmax	output three classes	Healthy, partial and rupture
Total parameters	179,075	

**Table 4:** List of selected real-time augmentation with arguments and their description.

Sr.No	Augmentation Arguments	Description
1.	featurewise_center	Set input mean to 0 over the dataset
2.	featurewise_std_normalization	Divide inputs by standard deviation of dataset
3.	zca_epsilon = 1 × 10^−6^	Epsilon for Zero-phase whitening (ZCA) whitening
4.	fill mode = ‘nearest’	Set mode for filling points outside the input boundaries
5.	horizontal flip = True	Randomly flip images horizontally
6.	vertical flip = True	Randomly flip images vertically

**Table 5:** The ResNet-14 CNN test loss and test accuracy.

ResNet-14 CNN Model Tested Approaches	Test Loss	Test Accuracy
Without class balancing and data augmentation (5-fold cross-validation)	1.294	0.805
Without class balancing but data augmentation (5-fold cross-validation)	1.089	0.774
Class balancing and without data augmentation (random splitting)	0.537	0.884
Class balancing and data augmentation (random splitting)	0.526	0.895
Class balancing and data augmentation (3-fold cross-validation)	0.533	0.895
Class balancing and data augmentation (5-fold cross-validation)	0.466	0.919

**Table 6:** Performance Metrics of model ResNet convolutional neural network (CNN).

Evaluation Metrics of ResNet-14 CNN					
	Multi Classes				
Approaches	Evaluation	Healthy Tear	Partial Tear	Full Torn	Average
Without class balancing and data augmentation (5-fold cross-validation)	Precision	0.85	0.57	0.57	0.663
	Sensitivity	0.96	0.39	0.22	0.523
	F1-Score	0.90	0.47	0.31	0.563
	Specificity	0.78	0.86	0.95	0.863
	Accuracy	0.81			
	AUC	0.87	0.81	0.91	0.863
Without class balancing with data augmentation (5-fold cross validation)	Precision	0.83	0.47	0.47	0.590
	Sensitivity	0.94	0.29	0.22	0.483
	F1-Score	0.88	0.36	0.30	0.513
	Specificity	0.70	0.78	0.96	0.813
	Accuracy	0.77			
	AUC	0.83	0.76	0.91	0.833
Hybrid class balancing without data augmentation (Random Splitting)	Precision	0.87	0.81	0.96	0.880
	Sensitivity	0.85	0.79	0.99	0.877
	F1-score	0.86	0.80	0.98	0.880
	Specificity	0.90	0.92	0,99	0.910
	Accuracy	0.88			
	AUC	0.96	0.95	0.99	0.967
Hybrid class balancing with data augmentation (random splitting)	Precision	0.89	0.84	0.94	0.890
	Sensitivity	0.86	0.81	0.99	0.887
	F1- score	0.88	0.83	0.97	0.893
	Specificity	0.91	0.92	0.99	0.940
	Accuracy	0.90			
	AUC	0.97	0.96	0.99	0.973
Hybrid class balancing with data augmentation (3-fold cross validation)	Precision	0.90	0.83	0.94	0.890
	Sensitivity	0.87	0.80	0.99	0.887
	F1- score	0.88	0.82	0.97	0.890
	Specificity	0.91	0.92	0.99	0.940
	Accuracy	0.90			
	AUC	0.97	0.94	0.99	0.967
Hybrid class balancing with data augmentation (5-fold cross validation)	Precision	0.92	0.87	0.96	0.917
	Sensitivity	0.89	0.87	0.99	0.917
	F1-score	0.90	0.87	0.98	0.917
	Specificity	0.93	0.92	0.99	0.947
	Accuracy	0.92			
	AUC	0.98	0.97	0.99	0.980

**Table 7:** Comparison state of the art works with our proposed model.

Author, Year	Model	Dataset	Target Output	Evaluation				
				Accuracy	Sensitivity	Precision	Specificity	AUC
Štajduhar et al. [23]	HOG+ Linear-kernel SVM (k = 10)	KneeMRI 917 (exams)	partial tear	-	-	-	-	0.894
			ruptured tear	-	-	-	-	0.943
Bien et al., 2018 [27]	AlexNet	MRNet 1370 exams	ACL tear	0.867	0.759	-	0.968	0.965
			abnormal	0.850	0.879	-	0.714	0.937
			meniscus tear	0.725	0.892		0.741	0.847
	Logistic Regression	KneeMRI 917 exam	partial tear, ruptured tear	-	-	-	-	0.911
Chang et al., 2019 [33]	Dynamic patch + ResNet	260 MRI coronal volumes	partial AC, full torn	0.967	1.00	0.938	0.933	-
Liu et al., 2019 [34]	VGG16	sagittal MR 175 (exams)	full thickness ACL tear, Intact ACL	-	0.92	-	0.92	0.95
	DenseNet			-	0.96	-	0.96	0.98
	Alex Net			-	0.89	-	0.88	0.90
Namiri et al., 2019 [39]	2D CNN 3D CNN	NIH MRI 1243 (exams)	Intact ACL	-	0.22 0.89	-	0.90 0.88	-
	2D CNN 3D CNN		Partial tear	-	0.75 0.25	-	1.00 0.92	-
	2D CNN 3D CNN		Full tear	-	0.82 0.76	-	0.94 1.00	-
Zhang et al., 2020 [40]	3D DenseNet	sagittal MR 408 (exams)	ACL tears Intact ACL	0.957 0.943 0.899	0.976 0.952 0.912	0.940 0.952 0.869	0.944 0.909 0.886	0.960 0.946 0.859
	ResNet							
	VGG16							
Irmakci et al., 2020 [41]	AlexNet	MRNet 1370 exams	abnormal	0.8583	0.978	-	0.400	0.891
			ACL tear	0.833	0.685	-	0.954	0.938
	ResNet-18		abnormal	0.825	0.968	-	0.280	0.811
			ACL tear	0.866	0.777	-	0.939	0.954
	GoogleLeNet		abnormal	0.833	0.978	-	0.280	0.909
			ACL tear	0.808	0.666	-	0.924	0.890
Tsai et al., 2020 [42]	EfficientNet	MRNet 1370	abnormal	0.917	0.968	-	0.72	0.941
			ACL tear	0.904	0.923	-	0.891	0.960
	ELNet 5 -fold	KneeMRI 917 exams	ruptured ACL	-	-	-	-	0.913
Proposed Customized ResNet-14 5-fold cross-validation		KneeMRI 917 exams	ACL Intact	0.92	0.89	0.92	0.93	0.98
			partial tear	0.91	0.87	0.87	0.92	0.97
			ruptured	0.93	0.99	0.96	0.99	0.99

## Data Availability

We are using this dataset in our work from Clinical Hospital Centre Rijeka, under reference [23].
